# Urban Environmental Barriers and Facilities to Mobility and Participation for Older Mobility Device Users

**DOI:** 10.1093/geroni/igaa057.1540

**Published:** 2020-12-16

**Authors:** W Ben Mortenson, Bill Miller, Atiya Mahmood, Francois Routhier, Delphine Labbe

**Affiliations:** 1 University of British Columbia, Vancouver, British Columbia, Canada; 2 University of British Columbia, Vancouver, Canada; 3 Simon Fraser University, Vancouver, British Columbia, Canada; 4 University of Laval, Quebec City, Quebec, Canada; 5 University of Illinois at Chicago, Chicago, Illinois, United States

## Abstract

Many people use mobility devices to get around. Unfortunately, these mobility device users frequently encounter environmental features and social practices that restrict mobility and social participation. For example, barriers in the built environment can exclude mobility devices users from certain spaces. They also report experiencing discrimination and stigma in the community. However, much of the research in this area has not examined the experiences of older mobility device users in a holistic manner. The purpose of our study was to explore the barriers and facilitators of mobility and participation among people who use wheeled mobility devices. This mixed-methods project used multiple participatory research methods including qualitative interviews, participant-led, community environmental audits, photovoice, mobility tracking using global positioning satellite data and building accessibility audits of participant nominated buildings. We used standardized tools to measure participants’ perceived, physical functioning, anxiety and depression, mobility and mobility device confidence among device users living. The study included 104 participants (64 from the Metro Vancouver and 41 from Quebec City). The primary mobility devices used included manual and power wheelchairs, mobility scooters, canes, crutches and walkers. On average, participants were 58 years of age and 53% were female. Our analysis revealed four main themes: 1) wayfinding challenges; 2) barriers and facilitators in the built environment; 3) the influence of social practices; and 4) temporal and climatic fluctuations. Our findings identified policies and changeable features in the built and social environment that restrict accessibility, which could be remedied by working collaboratively with municipalities and service providers.

